# Supplementary material for: DMRforPairs: identifying Differentially Methylated Regions between unique samples using array based methylation profiles
Source: BMC Bioinformatics. 2014 May 15;15:141. doi: 10.1186/1471-2105-15-141 (PMC4046028; doi:10.1186/1471-2105-15-141)
Supplement: Additional file 2 — DMRforPairs output for the comparison of A431-MCF7 and NA17018-NA17105. Please start from the HTML files in each folder. Available via the BMC Bioinformatics website. [file 1471-2105-15-141-S2.zip › 1394847754114233_MOESM2_ESM/A431_MCF7/figures/23461.pdf]

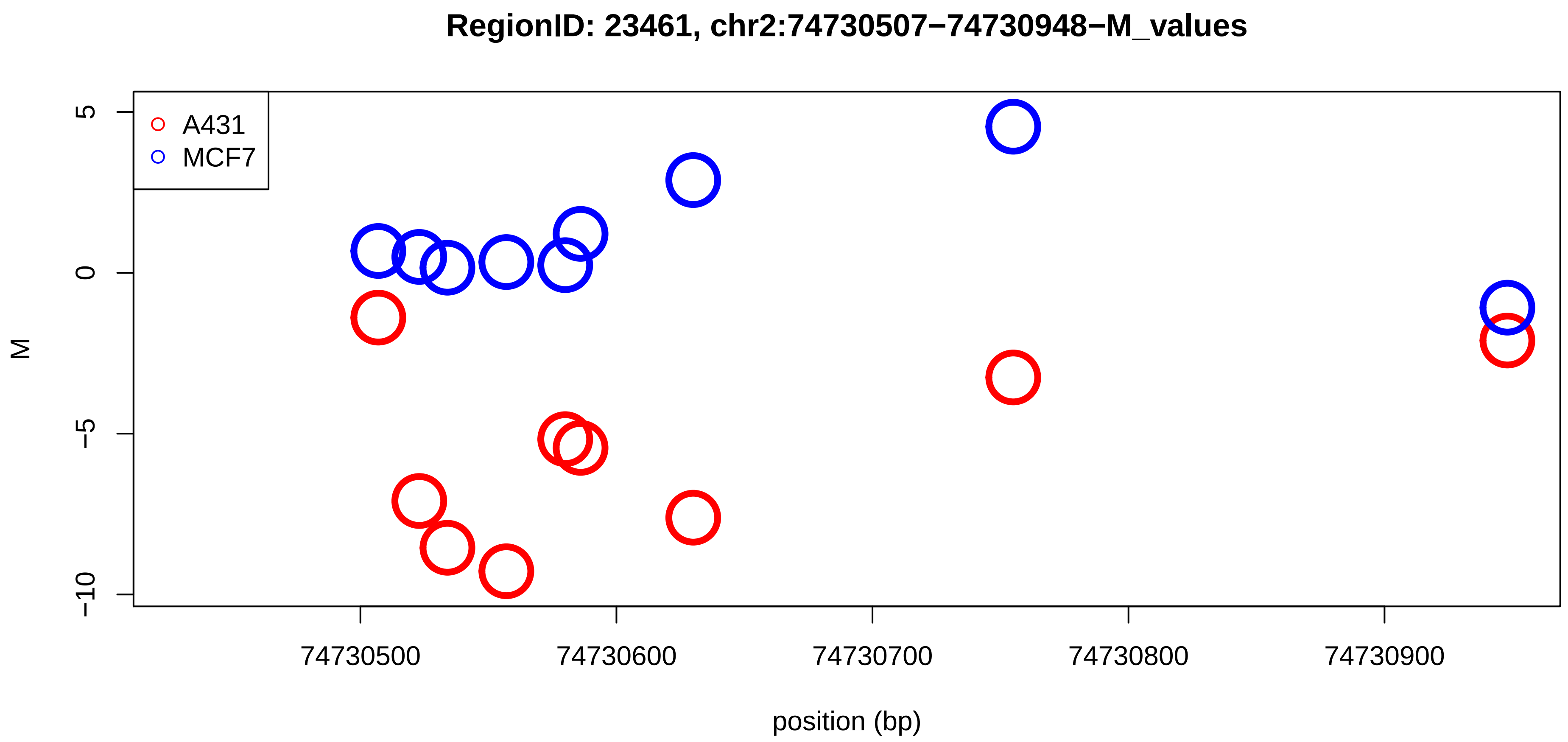

RegionID: 23461, chr2:74730507−74730948−M_values
A431
MCF7
M
5
0
−5
−10
74730500
74730600
74730700
74730800
74730900
position (bp)

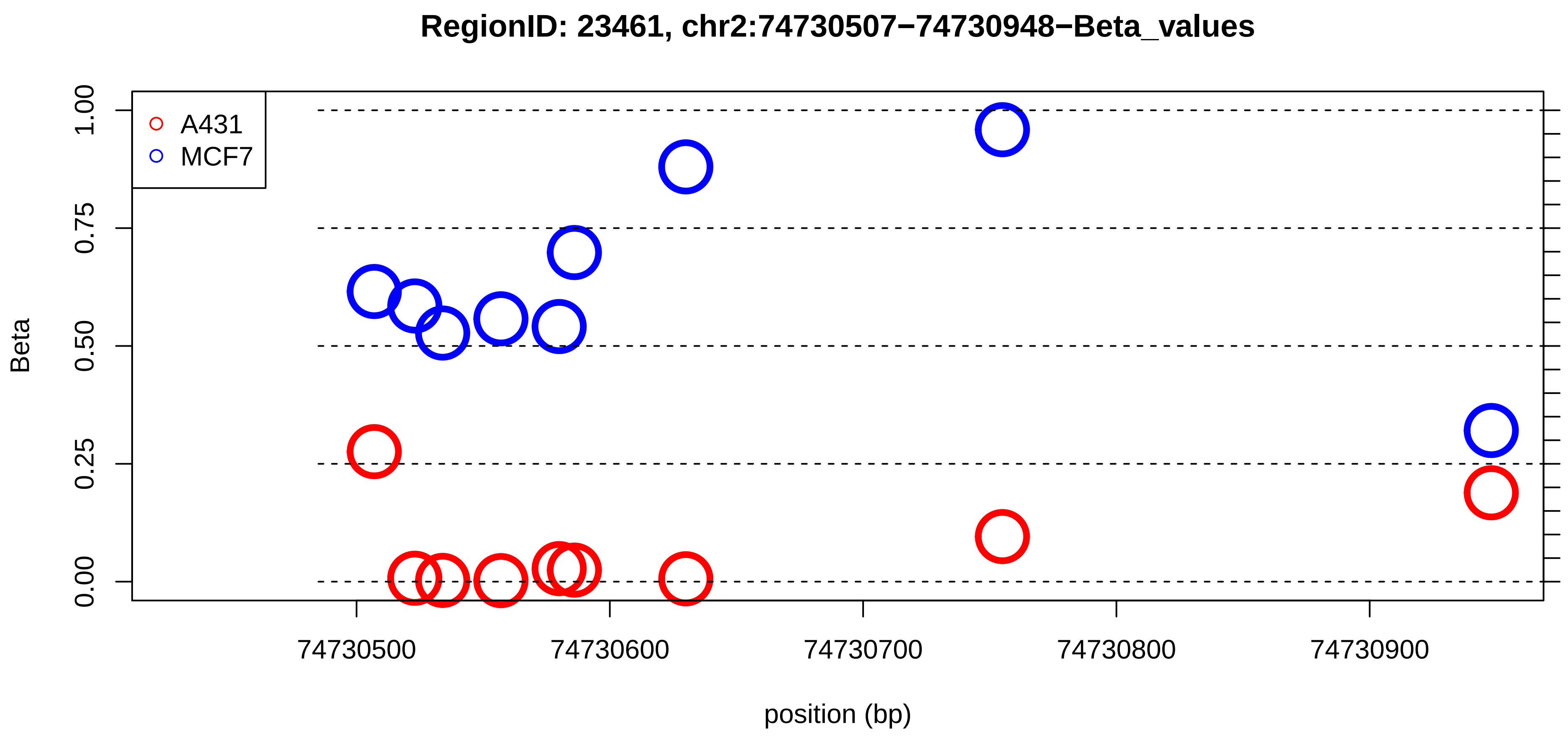

RegionID: 23461, chr2:74730507−74730948−Beta_values
A431
MCF7
Beta
1.00
0.75
0.50
0.25
0.00
74730500
74730600
74730700
74730800
74730900
position (bp)

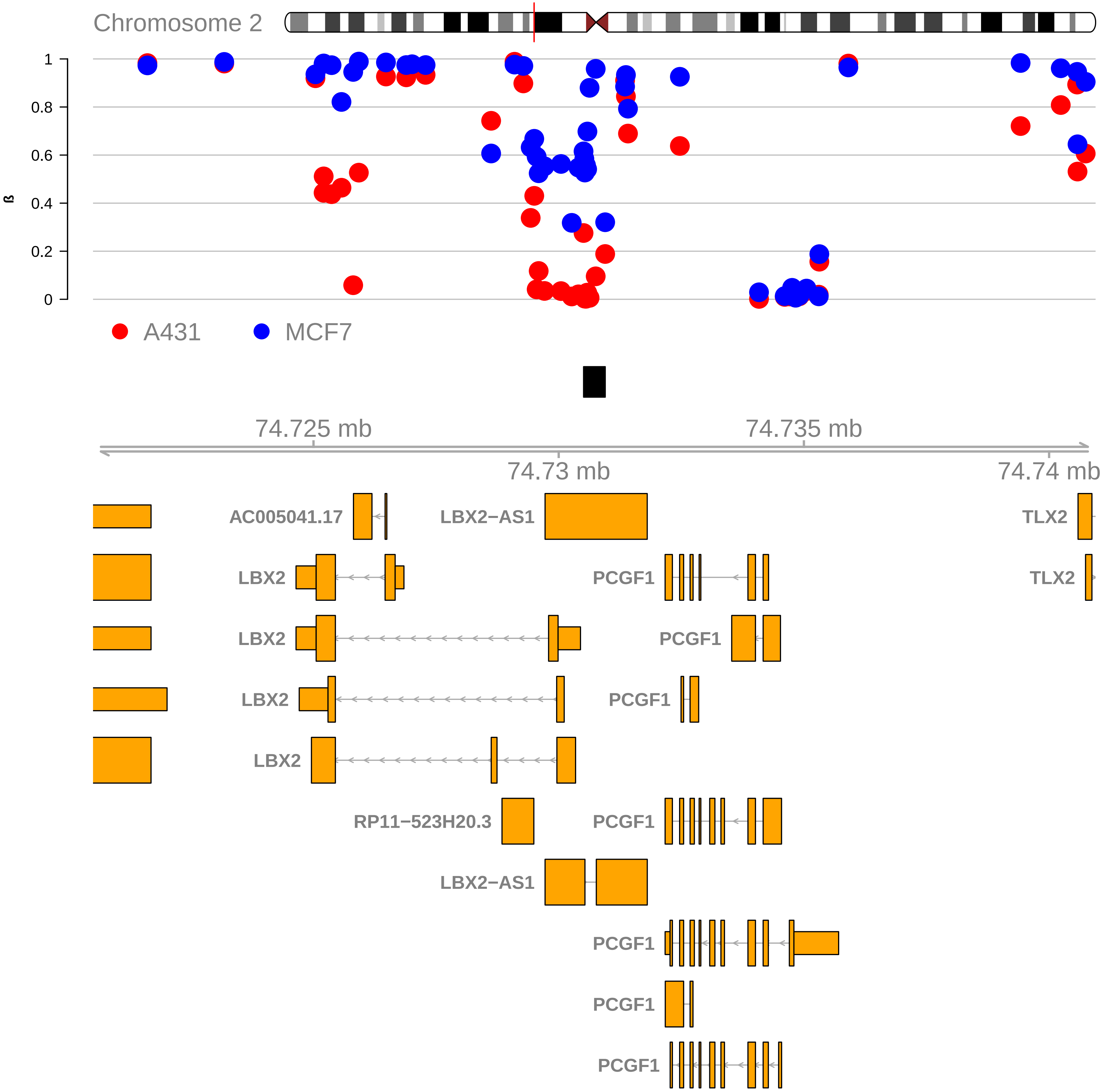

Chromosome 2
1
0.8
0.6
0.4
0.2
0
β
A431
MCF7
74.725 mb
74.735 mb
74.73 mb
74.74 mb
AC005041.17
LBX2−AS1
TLX2
LBX2
PCGF1
TLX2
LBX2
PCGF1
LBX2
PCGF1
LBX2
RP11−523H20.3
PCGF1
LBX2−AS1
PCGF1
PCGF1
PCGF1
